# Predictors of the response to phosphodiesterase-5 inhibitors in pulmonary arterial hypertension: an analysis of the Spanish registry

**DOI:** 10.1186/s12931-023-02531-1

**Published:** 2023-09-15

**Authors:** Agustin R. Garcia, Isabel Blanco, Lluis Ramon, Jesús Pérez-Sagredo, Francisco J. Guerra-Ramos, Clara Martín-Ontiyuelo, Olga Tura-Ceide, Francisco Pastor-Pérez, Pilar Escribano-Subías, Joan A. Barberà

**Affiliations:** 1 Department of Pulmonary Medicine, Hospital Clínic-IDIBAPS, University of Barcelona, Barcelona, Spain; 2 Biomedical Research Networking Center on Respiratory Diseases (CIBERES), Madrid, Spain; 3 Department of Pulmonary Medicine, Hospital El Bierzo, León, Spain; 4 Department of Pulmonary Medicine, Complejo Hospitalario Universitario Insular-Materno Infantil, Las Palmas, Spain; 5 Cardiology Department, Hospital Clínico Universitario Virgen de la Arrixaca, Murcia, Spain; 6 Pulmonary Hypertension Unit, Cardiology Department, Hospital Universitario 12 de Octubre, Madrid, Spain; 7 Biomedical Research Networking Center on Cardiovascular Diseases (CIBERCV), Madrid, Spain

**Keywords:** Pulmonary arterial hypertension, Vasodilator agents, Phosphodiesterase-5 inhibitors, Sildenafil citrate, Tadalafil, Treatment outcome

## Abstract

**Background:**

Achieving and maintaining a low-risk profile is associated with favorable outcome in pulmonary arterial hypertension (PAH). The effects of treatment on risk profile are variable among patients.

**Objective:**

To Identify variables that might predict the response to treatment with phosphodiesterase-5 inhibitors (PDE-5i) in PAH.

**Methods:**

We carried out a cohort analysis of the Spanish PAH registry in 830 patients diagnosed with PAH that started PDE5i treatment and had > 1 year follow-up. 644 patients started PDE-5i either in mono- or add-on therapy and 186 started combined treatment with PDE-5i and endothelin receptor antagonist (ERA). Responders were considered when at 1 year they: (1) were alive; (2) did not present clinical worsening; and (3) improved European Society of Cardiology/European Respiratory Society (ESC/ERS) risk score or remained in low-risk. Univariate and multivariate logistic regression models were used to analyze variables associated with a favorable response.

**Results:**

Two hundred and ten patients (33%) starting PDE-5i alone were classified as responders, irrespective of whether it was mono- or add-on therapy. In addition to known predictors of PAH outcome (low-risk at baseline, younger age), male sex and diagnosis of portopulmonary hypertension (PoPH) or HIV-PAH were independent predictors of favorable response to PDE-5i. Diffusing capacity for carbon monoxide (DLco) ≤ 40% of predicted was associated with an unfavorable response. When PDE-5i were used in upfront combination, 58% of patients were responders. In this group, diagnosis of idiopathic PAH (IPAH) was an independent predictor of favorable response, whereas connective tissue disease-PAH was associated with an unfavorable response.

**Conclusion:**

Male sex and diagnosis of PoPH or HIV-PAH are predictors of favorable effect of PDE-5i on risk profile when used as mono- or add-on therapy. Patients with IPAH respond more favorably to PDE-5i when used in upfront combination. These results identify patient profiles that may respond favorably to PDE-5i in monotherapy and those who might benefit from alternative treatment strategies.

**Supplementary Information:**

The online version contains supplementary material available at 10.1186/s12931-023-02531-1.

## Background

Pulmonary arterial hypertension (PAH) is a progressive disease leading to increased pulmonary vascular resistance, right ventricular (RV) failure and eventually death. Despite survival has improved with current treatment approach, the disease remains incurable [1, 2]. Assessment of mortality risk is an essential step in guiding PAH treatment, both at diagnosis and during follow-up [3]. Disease risk is usually evaluated using a multidimensional stratification according to clinical, exercise, imaging, biologic and hemodynamic variables with known prognostic significance [3]. The treatment goal is reaching and maintaining a low risk profile, although improving risk score as effect of treatment is also associated with better prognosis [4, 5].

The effect of targeted PAH therapy on modifying the risk profile among patients is diverse and unpredictable. The variability in the response to treatment is even higher depending on the type of drug [6, 7]. Furthermore, different clinical phenotypes have been identified among patients with PAH that are associated with different response to treatment [8, 9]. Recent clinical guidelines recommend initial monotherapy with a phosphodiesterase-5 inhibitor (PDE-5i) or an endothelin receptor antagonist (ERA) in patients with PAH and associated cardiopulmonary comorbidities [3]. In addition to comorbidities, identifying clinical traits that are associated with a favorable response to treatment will allow physicians to individualize the treatment approach.

Phosphodiesterase-5 inhibitors have been extensively used in the treatment of PAH and currently conform the first therapeutic option in PAH with low or intermediate risk, either as monotherapy in patients with comorbidities or in combination with an ERA in patients without comorbidities [3]. Sildenafil and tadalafil produce significant vasodilation and have antiproliferative effects by impeding the catabolism of cyclic guanosine monophosphate (cGMP), thereby enhancing nitric oxide (NO) signaling [10, 11]. Recently, it has been shown that replacing PDE-5i with a soluble guanylate cyclase (sGC) stimulator, which also acts on the NO signaling pathway, improves the clinical profile in patients with PAH [12]. Factors that might predict the response to PDE-5i have not been identified. Accordingly, the current study was aimed to identify in patients with PAH, demographic and clinical variables that could predict the effect of treatment with PDE-5i on their risk profile.

## Study design and methods

### Patient population

**Fig. 1:**
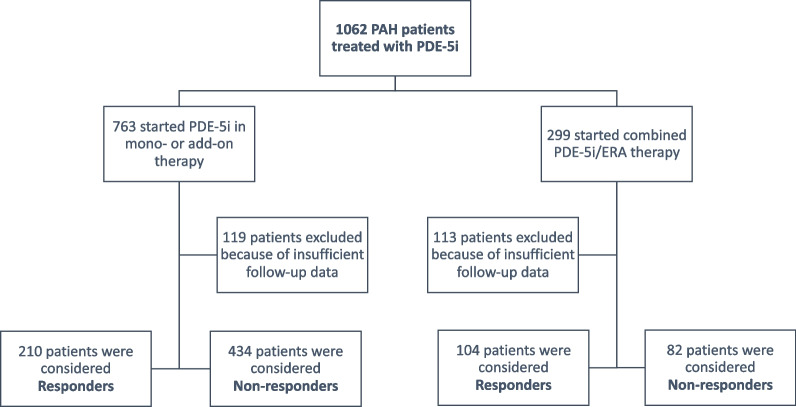
Patient disposition. Patients included in the Spanish PAH Registry (REHAP) exposed to phosphodiesterase-5 inhibitors (PDE-5i) were analyzed. Predictors of the response to treatment in patients initiating PDE-5i in monotherapy or as add-on treatment were analyzed and compared with those initiating PDE-5i in combination with an endothelin receptor antagonist (ERA)

We analyzed patients diagnosed with PAH from the Spanish Registry (REHAP) who initiated treatment with a PDE-5i either in monotherapy or as add-on therapy, between July 2007 and September 2019, were followed-up for at least 12 months, and had sufficient information available to evaluate the response to treatment. One thousand sixty-two patients treated with a PDE-5i were extracted from the REHAP. Among the 763 patients who started treatment with a PDE-5i either as a monotherapy or as add-on treatment, 119 were excluded because of insufficient data for risk stratification. The final study cohort comprised 644 patients (Fig. 1). Two hundred ninety-nine patients started combined therapy with PDE-5i and an ERA and were used as comparison group. Among them, 113 patients were excluded because lack of data for risk stratification. The final combined treatment group comprised 186 patients.

Patients were classified as responders to PDE-5i if at 12 months they: (1) were alive and free of lung transplant, (2) did not suffer clinical worsening events leading to treatment change, and (3) improved their risk score or remained in low-risk profile according to the ESC/ERS four-strata risk table [3]. Otherwise, patients were considered as non-responders to PDE-5i treatment. A treatment change was considered if: (1) an ERA or a prostanoid was added at least 60 days after starting PDE-5i treatment, or (2) PDE-5i treatment was interrupted more than 30 days after initiation or switched to riociguat, an ERA or a prostanoid.

### Risk stratification

We computed the individual risk score using the four-strata risk assessment and cut-off values proposed by Hoeper et al. [13], as recommended by the ERS/ESC guidelines for follow-up assessment [3]. The mean score was calculated by dividing the sum of all individual variable scores (graded from 1 to 4) by the number of variables. The result was rounded off to the nearest integer to define the risk category: 1 = low, 2 = intermediate-low, 3 = intermediate-high, and 4 = high risk. The risk score was calculated at baseline, before starting PDE-5i treatment, and at 1-year follow-up. For the 1-year follow-up assessment we considered measurements obtained between 9 and 18 months after starting PDE-5i treatment.

### Statical analysis

Continuous variables are given as mean value and standard deviation and categorical variables as total number and percentages. Logistic regression analyses were used to evaluate the effects of baseline characteristics on outcome. Univariate logistic regression models were fitted for each of the potential predictors. A p value < 0.05 was used to screen for covariates. Backward stepwise selection algorithms were used to select covariates to be included in the multivariate logistic regression model. At each step, the least significant variable was discarded from the model until reaching a p value of 0.1, independent covariates with a p below this value remained in the final model. Odds ratio and 95% confidence interval were calculated. Receiver operating characteristic (ROC) analyses were used to establish the optimal cutoff value for predicting the outcome.

To evaluate whether the predictors of the response to treatment were the same when the PDE-5i was used in upfront combination with an ERA, which became the standard of care since 2015 [3], the analysis was reproduced in the group of patients of the REHAP registry who started combined treatment with PDE-5i and ERA.

The statistical analysis was performed using R for Windows (version 4.0.4, R Project for Statistical Computing, Vienna, Austria).

## Results

According to our grouping strategy, among the 644 patients with PAH starting PDE-5i in monotherapy or as add-on treatment, 210 (33%) were considered responders and 434 (67%) non-responders (Fig. 1). Criteria for considering responders to PDE-5i treatment were: improvement in risk category (n = 116) and remaining in low risk (n = 94). Causes for being classified as non-responder were: death (n = 85), occurrence of clinical events leading to treatment change (n = 197), worsening in risk category (n = 38), and remaining in intermediate-low (n = 78), intermediate-high (n = 32) or high-risk (n = 3) category. Four hundred thirty-two patients were treatment-naïve who started the PDE-5i in monotherapy and 212 were prevalent patients in whom the PDE-5i was added to ongoing therapy. Patient demographics, general characteristics and risk variables of the responders and the non-responders groups are shown in Table 1. The proportion of treatment-naïve (incident) patients starting PDE-5i in monotherapy or prevalent patients starting PDE-5i as add-on therapy was similar among responders and non-responders**.** Responders were younger (50 ± 16 vs 55 ± 16 years, p < 0.001) and in a greater proportion male (41% vs 30%, p = 0.011) compared to non-responders. Pulmonary hemodynamics were similar in both groups, whereas in the responders the baseline risk profile was low in a greater proportion of patients (44% vs 17%, p < 0.001), with lower functional class (FC I-II 60% vs 33%), greater distance covered in the 6 min walk test (434 ± 123 vs 361 ± 117 m p < 0.001) and lower NT-proBNP (1004 ± 1998 vs 2309 ± 3205 pg/ml, p < 0.001). The proportion of patients in low risk profile was similar among incident and prevalent patients (28% vs 26%, respectively; p = 0.21).

**Table 1:** Baseline characteristics of patients initiating PDE-5i treatment in monotherapy or as add-on therapy

	Responders​ (n = 210)​	Non-responders​ (n = 433)​	*P* value
Age, yrs​	50 ± 16​	55 ± 16​	< 0.001*
Male sex	41%	30%	0.011*
Weight, Kg​	69 ± 15	68 ± 16	0.919
Height, cm​	163 ± 10​	160 ± 10​	0.080*
PDE-5i started in monotherapy/add-on therapy	66%/34%	67%/33%	0.885
Systolic blood pressure, mmHg​	127 ± 21	128 ± 23​	0.565
Diastolic blood pressure mmHg​	73 ± 12​	74 ± 12​	0.929
Hearth rate, bpm​	75 ± 12​	78 ± 13​	0.031*
PAH etiology			
Idiopathic	25%​	33%​	0.085
Drugs and toxins	2%​	2%​	1.000
Connective tissue disease	25%​	26% ​	0.791
Portopulmonary hypertension	18%​	9%	< 0.01*
HIV	11%	4%​	< 0.01*
Congenital heart disease	19%​	14%​	0.121
PVOD	4%​	6%​	0.082
Pulmonary function			
DLco, % pred	57±19​	49±23	<0.01*
FVC, % pred	86±18​	84±20​	0.350
FEV1, % pred	81±17​	80±19​	0.560
FEV1/FVC, %	75±7​	75±10​	0.870
Hemodynamic measurements			
mPAP, mmHg	48±13​	49±13​	0.640
PVR, WU	9.6±4.9​	10.1±5.7​	0.340
CI, L/min/m^2^	2.5±0.8​	2.6±0.8​	0.520
RAP, mmHg	8.8±5.1​	9.7±5.8​	0.120
SvO_2_, %	65±10​	65±9​	0.890
Prognosis determinants and baseline risk			
FC I-II/FC III-IV	60%/40%​	33%/67%	< 0.001*
6-MWD, m	434±123​	361±117	< 0.001*
NT-proBNP, pg/mlL	1004±1998​	2309±3205	< 0.001*
RA area, cm^2^	20.8 ± 7.5​	23.8 ± 7.2	0.029*
Low risk, n (%)	44%	17%​	< 0.001*
Intermediate risk, n (%)	51%	73%​	< 0.001*
High risk, n (%)	5%	10%​	< 0.001*

Variables are mean ± SD or percentage into each group. *p < 0.05

*PDE-5i *phosphodiesterase-5 inhibitor, *HIV *human immunodeficiency virus, *PVOD *pulmonary veno-oclusive disease, *DLco* diffusing capacity for carbon monoxide, *FVC *forced vital capacity, *FEV*_1_ forced expiratory volume in the first second, *mPAP* mean pulmonary artery pressure, *PVR* pulmonary vascular resistance, *WU* Wood units, *CI* cardiac index, *RAP* right atrial pressure, *S*_v_*O*_2_ mixed-venous oxygen saturation, *FC* New York Heart Association functional capacity, *6-MWD* Six-min walking test distance, *NT-proBNP* N-terminal pro-brain natriuretic peptide, *RA* right atrium

### Univariate analysis

Table 2 shows the results of the univariate analysis of variables predictive of a favorable response to PDE-5i treatment. Male sex and younger age (≤ 50 years) were significantly associated with a favorable response to PDE-5i. Regarding PAH subtypes, patients with portopulmonary hypertension (PoPH) or HIV-PAH were also more likely to respond favorably to PDE-5i, whereas patients with idiopathic PAH (IPAH) showed a trend to be non-responders. A low-risk score at baseline and risk determinants with values in low-risk range were also associated with a favorable response to PDE-5i. Being on background PAH therapy was not related to the response to PDE-5i.

**Table 2:** Univariate and multivariate logistic regression analyses of predictors of a favorable response to treatment with PDE-5i in mono- or add-on therapy

	Univariate analysis		Multivariate analysis	
	OR (95% CI)	*P* value	OR (95% CI)	*P* value
Age ≤ 50 years	1.49 (1.07–2.08)	0.017*	1.41 (0.98–2.00)	0.052
Male sex	1.59 (1.12–2.24)	0.009*	1.38 (0.96–1.97)	0.081
PDE-5i in monoteraphy	1.04 (0.73–1.48)	0.815		
Low risk category	5.55 (2.70–12.5)	< 0.001*	7.69 (2.38–25.0)	< 0.001*
PAH etiology				
IPAH	0.71 (0.49–1.03)	0.070		
CTD-PAH	1.07 (0.73 –1.57)	0.717		
PoPH	2.04 (1.25–3.33)	0.004*	1.88 (1.14–3.12)	0.013*
HIV-PAH	2.63 (1.36–5.00)	0.002*	2.04 (1.05–4.00)	0.035*
Hemodynamic measurements				
mPAP, mmHg	1.00 (0.98–1.01)	0.643		
PVR, WU	1.02 (0.98–1.05)	0.336		
CI, L/min/m^2^	1.09 (0.84–1.41)	0.515		
RAP, mmHg	1.03 (0.99–1.07)	0.121		
S_v_O_2_, mmHg	0.99 (0.97–1.02)	0.893		
Functional capacity and pulmonary function				
WHO FC I–II	7.14 (3.06–16.1)	< 0.001*		
6MWT $\ge$ 440m	3.42 (2.27–5.15)	< 0.001*		
DLco ≤ 40% pred	0.31 (0.19–0.51)	< 0.001*	0.25 (0.11–0.57)	0.001*
Biomarkers and echocardiography				
NT-proBNP ≤ 300 pg/mlL	3.03 (1.81–5.00)	< 0.001*		
RA area ≤ 20 cm^2^	1.11 (1.01–1.25)	0.029*		

^*^p < 0.05

*PDE-5i *phosphodiesterase-5 inhibitor, *PAH *idiophatic pulmonary arterial hypertension, *CTD* connective tissue disease, *PoPH* portopulmonary hypertension, *HIV-PAH* HIV associated pulmonary arterial hypertension, *mPAP* Mean pulmonary artery pressure, *CI* cardiac index, *PVR *Pulmonary vascular resistance, *WU *Woods Units, *RAP *right atrial pressure, *S*_v_*O*_2_ mixed venous oxygen saturation, *DLco* Diffusing capacity for carbon monoxide, *WHO FC *functional classification, *6MWD *Six-min walking test distance, *NT-proBNP* N-terminal pro-brain natriuretic peptide, *RA area*  right atrium area

### Multivariate analysis

Male sex, age ≤ 50 years and a lower risk profile at baseline were independent predictors of favorable response, while diffusing capacity for carbon monoxide (DLco) ≤ 40% of predicted value was an independent predictor of non-response. Patients with PoPH or HIV-PAH also had greater likelihood to respond favorably to PDE-5i in the multivariate analysis (Table 2).

### Response to PDE-5i in upfront combination

In the group of patients starting PDE-5i and ERA combined therapy, 104 (56%) were considered responders and 82 (44%) non-responders (Fig. 1). Patient demographics, general characteristics and risk variables of the responders and the non-responders groups are shown in Table 3**.** In these patients age ≤ 50 years was also associated with a favorable response to treatment, whereas sex was unrelated to the treatment response (Table 4). Regarding baseline risk determinants, only 6-MWD ≥ 440 m and FC I-II were associated to a greater likelihood of favorable response (Table 4). In the univariate analysis, patients with IPAH were more likely to respond favorably, whereas a diagnosis of PAH associated with connective tissue disease (CTD-PAH) was associated with lack of response to combined treatment with PDE-5i and ERA. Since the number of patients with HIV-PAH and PoPH starting combination therapy was reduced, it was not possible to analyze the effect of these diagnoses on treatment response.

**Table 3:** Baseline characteristics of patients initiating combination therapy of PDE-5i and ERA

	Responders (n = 104)	Non-responders (n = 82)	*P* value
Age, yrs	47 ± 15	55 ± 16	< 0.001*
Male sex	32%	30%	0.996
Weight, Kg	68.5 ± 16	68 ± 14	0.980
Height, cm	163 ± 10	162 ± 10	0.582
PDE-5i started in monotherapy/add-on therapy	–	–	
Systolic blood pressure, mmHg	121 ± 18	124 ± 26	0.513
Diastolic blood pressure mmHg	72 ± 11	71 ± 14	0.718
Hearth rate, bpm	82 ± 16	82 ± 13	0.996
PAH etiology			
Idiopathic	52%	27%	< 0.01*
Drugs and toxins	1%	1%	1.000
Connective tissue disease	19%	42%	< 0.01*
Portopulmonary hypertension	7%	5%	0.764
HIV	6%	0%	0.041*
Congenital heart disease	13%	14%	0.994
PVOD	6%	13%	0.137
Pulmonary function			
DLco, % pred	55 ± 19	45 ± 20	< 0.01*
FVC, % pred	89 ± 15	82 ± 17	0.040*
FEV1, % pred	84 ± 17	80 ± 18	0.071
FEV1/FVC, %	74 ± 13	76 ± 9	0.643
Hemodynamic measurements			
mPAP, mmHg	56 ± 15	52 ± 17	0.072
PVR, WU	13.4 ± 5.9	10.6 ± 5.4	< 0.01*
CI, L/min/m^2^	2.3 ± 0.7	2.6 ± 0.9	0.050
RAP, mmHg	10.1 ± 5.4	10.2 ± 5.2	0.956
SvO_2_, %	61 ± 11	67 ± 6	< 0.01*
Prognosis determinants and baseline risk			
FC I-II/FC III–IV	24%/76%	31%/69%	0.125
6-MWD, m	394 ± 133	347 ± 116	0.023*
NT-proBNP, pg/mlL	2480 ± 3890	2318 ± 2880	0.654
RA area, cm^2^	22 ± 6	25 ± 7	0.113
Low risk, n (%)	64%	3%	< 0.001*
Intermediate risk, n (%)	36%	68%	< 0.001*
High risk, n (%)	0%	29%	< 0.001*

Variables are mean ± SD or percentage into each group. *p < 0.05

*PDE-5i* phosphodiesterase-5 inhibitor, *HIV* human immunodeficiency virus, *PVOD* pulmonary veno-oclusive disease; *DLco* diffusing capacity for carbon monoxide, *FVC* forced vital capacity, *FEV*_*1*_ forced expiratory volume in the first second, *mPAP* mean pulmonary artery pressure, *PVR* pulmonary vascular resistance, *WU* Wood units, *CI *cardiac index, *RAP* right atrial pressure, *S*_*v*_*O*_*2*_ mixed-venous oxygen saturation, *FC* New York Heart Association functional capacity, *6-MWD* six-minute walking test distance, *NT-proBNP* N-terminal pro-brain natriuretic peptide, *RA* right atrium

**Table 4:** Univariate and multivariate logistic regression analysis of predictors of a favorable response to upfront PDE-5i/ERA combined therapy

	Univariate analysis		Multivariate analysis	
	OR (95% CI)	*P* value	OR (95% CI)	*P* value
Age ≤ 50 years	2.77 (1.53–5.26)	< 0.001*	2.94 (1.31–6.67)	< 0.01*
Male sex	1.01 (0.53–1.92)	0.975		
Low risk category	3.03 (1.22–7.52)	0.017*	3.96 (1.23–12.81)	0.022*
PAH etiology				
IPAH	2.94 (1.56–5.55)	< 0.001*	3.03 (1.36–6.67)	< 0.01*
CTD-PAH	0.32 (0.17–0.63)	< 0.001*	0.41 (0.16–1.09)	0.074
Hemodynamic measurements				
mPAP, mmHg	1.00 (0.98–1.01)	0.643		
CI, L/min/m^2^	1.09 (0.84–1.41)	0.515		
PVR, WU	1.02 (0.98–1.05)	0.336		
RAP, mmHg	1.03 (0.99–1.07)	0.121		
S_v_O_2_, mmHg	0.99 (0.97–1.02)	0.893		
Functional capacity and pulmonary function				
DLco ≤ 40%	0.43 (0.20–0.91)	< 0.001*	0.29 (0.17–0.53)	< 0.01*
WHO FC I-II	2.90 (1.60–4.85)	< 0.001*		
6MWD $\ge$ 440 m	2.36 (1.13–4.90)	0.022*		
Biomarker and echocardiography				
NT-proBNP ≤ 300 pg/mL	1.49 (0.52–4.23)	0.454		
RA area ≤ 20cm^2^	1.63 (0.61–4.31)	0.325		

^*^p < 0.05

*PDE-5i *phosphodiesterase-5 inhibitor, *ERA* endothelin receptor antagonist, *IPAH *idiophatic pulmonary arterial hypertension, *CTD*  connective tissue disease, *mPAP* mean pulmonary artery pressure, *CI* cardiac index, *PVR* pulmonary vascular resistance, *WU* Wood Units, *RAP* right atrial pressure, *S*_*v*_*O*_*2*_ venous oxygen saturation, *DLco*  Diffusing capacity for carbon monoxide, *WHO FC* functional classification, *6MWD* Six-minute walking test distance, *NT-proBNP* N-terminal pro-brain natriuretic peptide, *RA area* right atrium area

In the multivariate analysis only age ≤ 50 years, low-risk profile at baseline and diagnosis of IPAH were independent predictors of a favorable response to initial combination therapy with PDE-5i and ERA (Table 4). In contrast, diagnosis with CTD-PAH and DLco ≤ 40% of predicted were independent predictors of poor response to combined treatment.

## Discussion

Our results show that in a real-life cohort only 33% of patients with PAH improved their risk profile when treated with a PDE-5i, either in monotherapy or as add-on treatment. In addition to well-known variables associated with better PAH outcomes, independent predictors of a favorable response to PDE-5i were male sex, younger age and a diagnosis of PoPH or HIV-PAH. On the other hand, DLco ≤ 40% of predicted value was associated with lack of improvement with PDE-5i treatment. In contrast, when a PDE-5i was used in upfront combination with an ERA, having a diagnosis of IPAH and younger age, were predictors of favorable effect on risk profile, whereas diagnosis of CTD-PAH and DLco ≤ 40% of predicted were associated with a poor response to combined treatment.

Achieving a low risk profile is a major treatment goal in PAH. In this analysis of the Spanish REHAP registry, only one third of patients improved their clinical status with PDE-5i treatment, either in mono or as add-on therapy, as assessed using a composite endpoint that included mortality, clinical worsening and change in ESC/ERS risk category. In the 2022 ESC/-ERS pulmonary hypertension guidelines it is recommended to start monotherapy in PAH patients with cardiorespiratory comorbidities [3], thereby raising the interest for identifying those patients that might be more suitable to a specific class of drug. Whereas, upfront combination therapy with PDE-5i and ERA is the standard of care in patients diagnosed with idiopathic, heritable, drug-associated and CTD-associated PAH without comorbidities [3], the benefit of this strategy remains unclear in PAH from other etiologies, where initial treatment with PDE-5i might be considered.

In our study, the proportion of patients achieving the favorable response criteria was similar in treatment-naïve and those who were already on background PAH-therapy, 32% and 36%, respectively. The impact of background treatment on the effect of a new targeted PAH drug on the risk profile has not been thoroughly evaluated. In a post-hoc analysis of the GRIPHON trial, Sitbon et al. [14] showed that the addition of selexipag had a beneficial effect on risk profile, regardless of whether they were on background therapy or not. Furthermore, using a composite endpoint similar to that used in the present study, Hoeper et al. [12] did not find differences in the response to switching from PDE-5i to riociguat between patients who were in combination therapy or those in monotherapy in the REPLACE study. Accordingly, preexisting treatment does not appear to exert substantial effect on the response to PDE-5i treatment.

In addition to well-known predictors of favorable outcome in PAH, namely younger age, and FC, 6-MWD and NT-proBNP within low-risk range, our study shows that PoPH and HIV-PAH subtypes are independent predictors of a favorable response to PDE-5i treatment. Long-term prognosis and evolution are diverse among PAH subtypes, and it is currently unclear the influence of different etiologies on the response to treatment [15]. Significant improvements in pulmonary hemodynamics, exercise endurance and biomarkers with PDE-5i treatment have been reported in both PoPH [16, 17] and HIV-PAH [18–20], although these patients have not been usually included in randomized clinical trials conducted in PAH. In previous analyses of the REHAP registry [21, 22], most patients with HIV-PAH and PoPH started treatment with oral monotherapy, including PDE-5i. Prior to the publication of the PORTICO trial with macitentan [23], PDE-5i have been extensively used for the treatment of PoPH, showing significant improvement in risk profile and survival previous to liver transplantation [16, 21, 24]. In liver disease, increased hepatic resistance and hyperdynamic changes are associated with a disturbed production and metabolism of NO [25, 26]. Nitric oxide donors, such as nitroglycerin [27] and isosorbide-5-mononitrate have shown to reduce both PVR and portal pressure [28] in liver cirrhosis. Conceivably, the favorable response to PDE-5i in PoPH might be related to an increased cGMP availability compensating the disturbed NO metabolism. In the same way, HIV proteins (Tat and gp120) induce the production of reactive oxygen species that might decrease endothelial NO synthase (eNOS) activity and NO availability in these patients [29]. In this scenario, PDE-5i might also compensate the reduced NO availability by blocking the hydrolysis of cGMP. Furthermore, it is also conceivable that the interaction of PDE-5i with antiretroviral drugs might explain, at least in part, the favorable response to PDE-5i in HIV-PAH [30]. Indeed, ritonavir, saquinavir and other protease inhibitors have an effect on cytochrome P450, CYP3A4 and CYP2C9, which are important in the metabolism of PDE-5i, leading to an increase in their plasma levels [3, 31, 32].

The multivariate analysis showed that women aged > 50 years and with DLco $\le$ 40% of predicted had lower likelihood to respond to PDE-5i in mono- or as add-on therapy. This observation is of interest since female sex has been associated with more favorable outcomes [33–35]. Our results showing a greater likelihood of response to PDE-5i in male patients concur with previous publications [36]. Sexual hormones influence plasma levels of vasoactive agents and the response to specific class of drugs [37]. Forte et al. [38] reported that men may be NO deficient and thus respond more favorably to PDE-5 inhibition, whereas Gabler et al. [39] showed higher levels of circulating endotelin-1 and greater clinical benefit from ERAs in women. Differences in the response to PDE-5i treatment between males and females may also rely on the underlying metabolism of NO [36, 38]. Indeed, irregular NO-mediated vasodilation, due to the presence of sGC independent pathways, has been shown in female animal models [40]. Therefore, underlying gender-specific, pathophysiological pathways and hormonal influence might explain a different response to PDE-5i in men and women. These disparities might have clinical implications. For instance, in men with IPAH and cardiopulmonary comorbidities, starting treatment with PDE-5i in monotherapy might be an appropriate option, in line with current guidelines [3].

A final observation is that patients starting treatment with PDE-5i in monotherapy or add-on therapy had greater effect when they were in a lower risk profile at baseline. Conversely, when patients started PDE-5i/ERA combined treatment, having markers of RV dysfunction (NT-proBNP and right atrial area) [41] in low risk range was unrelated to the response to treatment. Accordingly, in patients with RV dysfunction starting combined treatment would be more appropriate than starting monotherapy with a PDE-5i, even in those with associated comorbidities.

In this analysis of a real-life cohort, the proportion of patients with PAH considered responders to initial upfront combined therapy was 56%, much greater than in those who started treatment with PDE-5i alone, in line with the results of the AMBITION trial [42, 43]. Other studies also showed that up to 47% of patients have a favorable and sustained effect on risk profile after starting combined therapy [44, 45]. A recent analysis of the AMBITION population showed a higher rate of favorable response in patients receiving initial combination therapy, 77%, and identified female sex and a lower risk profile at baseline as predictors of favorable response [46]. The diagnosis of IPAH showed a trend to poor response to PDE-5i treatment alone, whereas it was a strong predictor of favorable response to upfront combination with PDE-5i and ERA. The fact that predictors of the response to PDE-5i differ when used alone or in combination with an ERA, suggests that the results we have obtained identify specific predictors of the response to this class of drug.

This study has some limitations. First, this is a retrospective analysis of a national registry without preestablished follow-up assessment and management criteria. Therefore, a sizeable number of patients did not have enough data for risk stratification at follow-up and clinical management might had been heterogenous, which might induce some bias in the obtained results, although this is inherent to analyses based on registry data. Second, the management and prognosis of the disease has changed considerably along time. Accordingly, we had to go back several years to analyze enough patients who had received PDE5 in monotherapy or as add-on treatment. Furthermore, due to changes in treatment practice we could not collect a contemporary cohort of patients receiving similar treatment regimen with PDE-5i that could be used as validation cohort. To overcome this limitation, we analyzed patients receiving upfront combination treatment with PDE-5i and ERA as comparative cohort, showing that the response profile is different when PDE-5i are used alone or in combination. Finally, we could not analyze the influence of cardiovascular comorbidities on the response to PDE-5i because these have not been prospectively included in the REHAP registry. Although, since the age of the patients in our study was younger than in other contemporary cohorts [47], it is conceivable that the prevalence of comorbidities and impact of cardiopulmonary comorbidities might be lower.

## Conclusions

In conclusion, this analysis of the Spanish REHAP registry shows that only one in three patients with PAH show a favorable response on risk profile with PDE-5i treatment. In addition to well established predictors of PAH outcome, diagnosis of PoPH or HIV-PAH and male sex emerge as specific predictors of a favorable effect of PDE-5i on risk profile. Conversely, the diagnosis of IPAH is associated with a favorable response to initial combination of PDE-5i and ERA. These results identify patient profiles that may respond favorably to PDE-5i monotherapy and those who might benefit from an alternative treatment strategy.

### Supplementary Information

**Additional file 1**: Investigators of the Spanish Pulmonary Arterial Hypertension Registry (REHAP) .

## Data Availability

The datasets used and/or analysed during the current study are available from the corresponding author on reasonable request.

## Abbreviations

6-MWD: 6-Minute walking distance
BNP: Brain natriuretic peptide
cGMP: Cyclic guanosine monophosphate
CI: Cardiac index
CTD: Connective tissue disease
DLco: Diffusing capacity for carbon monoxide
eNOS: Endothelial nitric oxide synthaset
ERA: Endothelin receptor antagonis
ERS: European Respiratory Society
ESC: European Society of Cardiology
FC: Functional class
HIV: Human immunodeficiency virus
IPAH: Idiopathic pulmonary arterial hypertension
NO: Nitric oxide
NT-proBNP: N-terminal pro b-type natriuretic peptide
PAH: Pulmonary arterial hypertension
PDE-5i: Phosphodiesterase-5 inhibitor
PH: Pulmonary hypertension
PoPH: Portopulmonary hypertension
RAA: Right atrium area
RAP: Right atrial pressure
REHAP: Spanish pulmonary arterial hypertension registry
ROC: Receiver operating characteristic
RV: Right ventricular
sGC: Soluble guanylate cyclase
S_v_O_2_: Mixed venous oxygen saturation
