# Supplementary material for: Predictors of the response to phosphodiesterase-5 inhibitors in pulmonary arterial hypertension: an analysis of the Spanish registry
Source: Respir Res. 2023 Sep 15;24:223. doi: 10.1186/s12931-023-02531-1 (PMC10503122; doi:10.1186/s12931-023-02531-1)
Supplement: Supplementary file 1 — Additional file 1: Investigators of the Spanish Pulmonary Arterial Hypertension Registry (REHAP) . [file 12931_2023_2531_MOESM1_ESM.doc]

**Investigators of the Spanish Pulmonary Arterial Hypertension Registry (REHAP)**

Álvarez Barredo M, Complejo Hospitalario Universitario Santiago de Compostela, A Coruña;

Angullo Gómez M, Hospital Clínico Universitario Virgen de la Victoria, Málaga;

Aurtenetxe Pérez A, Hospital Universitario Basurto, Bilbao;

Barbera JA, Hospital Clínic i Provincial de Barcelona, Barcelona;

Becerra Muñoz VM, Hospital Clínico Universitario Virgen de la Victoria, Málaga;

Bedate P, Hospital Universitario Central de Asturias, Asturias;

Blanco I, Hospital Clínic i Provincial de Barcelona, Barcelona;

Bravo Marqués R, Hospital Costa del Sol, Málaga;

Cadenas Menéndez S, Hospital Universitario de Salamanca (Clínico), Salamanca;

Callejas Rubio JL, Hospital Universitario Clínico San Cecilio, Granada;

Carmona Segovia AM, Hospital Clínico Universitario Virgen de la Victoria, Málaga;

Carrillo J, Hospital Universitario Rey Juan Carlos, Madrid;

Casado Moreno I, Hospital Universitario Virgen de las Nieves, Granada;

Castejón Pina N, Hospital Universitario Nuestra Señora de Candelaria, Santa Cruz de Tenerife;

Castro M , Hospital Álvaro Cunqueiro, Pontevedra;

Chamorro Fernández CI, Hospital Virgen de los Lirios, Alicante;

Cifrián JM, Hospital Universitario Marqués de Valdecilla, Cantabria;

Clavero Sánchez T, Hospital Universitario de Salamanca (Clínico), Salamanca;

Crémer Luengos D, Hospital Universitario Son Llàtzer, Islas Baleares;

del Prado Díaz S, Hospital Universitario Ramón y Cajal, Madrid;

Dobarro D, Hospital Álvaro Cunqueiro, Pontevedra;

Domingo Morera JA, Hospital Universitario Miguel Servet, Zaragoza;

Elías Hernández T, Hospital Universitario Virgen del Rocío, Sevilla;

Escribano SubíasP, Hospital Universitario 12 de Octubre, Madrid;

García Flores PI, Hospital Universitario Virgen de las Nieves, Granada;

García Hernández FJ, Hospital Universitario Virgen del Rocío, Sevilla;

Gonçalves Dos Santos Carvalho F, Hospital Universitari Germans Trias i Pujol, Barcelona;

González Segovia A, Hospital Universitario Puerta de Hierro, Madrid;

Guerra Ramos FJ, Complejo Hospitalario Universitario Insular-Materno Infantil, Las Palmas;

Hermida T, Hospital Universitario Central de Asturias, Asturias;

Jaimes Diaz SV, Hospital General Universitario de Valencia, Valencia;

Jara Palomares L, Hospital Universitario Virgen del Rocío, Sevilla;

Jiménez Arjona J, Hospital Universitario de Jerez de la Frontera, Cádiz;

Lacuey Lecumberri G, Hospital Universitario de Navarra, Navarra;

Lara Padrón A, Hospital Universitario de Canarias, Santa Cruz de Tenerife;

Lázaro Salvador M, Hospital Universitario de Toledo, Toledo;

López-Meseguer M, Hospital Universitario Vall d´Hebrón, Barcelona;

López Reyes R, Hospital Universitario y Politécnico La Fe, Valencia;

Luna López R, Hospital Central de la Defensa Gómez Ulla, Madrid;

Marín González M, Hospital Clínico Universitario de Valencia, Valencia;

Márquez Moreno JM, Hospital Universitario Jaén, Jaén;

Martínez García F, Hospital General Universitario Los Arcos del Mar Menor, Murcia;

Martínez Meñaca A, Hospital Universitario Marqués de Valdecilla, Cantabria;

Mazo Etxaniz FJ, Hospital Universitario Basurto, Bilbao;

Melendo Viu M, Hospital Álvaro Cunqueiro, Pontevedra;

Melero Ferrer J, Hospital General Universitario de Valencia, Valencia;

Mombiela T, Hospital Universitario Gregorio Marañón, Madrid;

Mora Cuesta VM, Hospital Universitario Marqués de Valdecilla, Cantabria;

Naranjo Velasco V, Hospital Universitario de Jerez de la Frontera, Cádiz;

Noris Mora M, Hospital Universitario Son Espases, Islas Baleares;

Núñez Ares A, Complejo Hospitalario Universitario de Albacete, Albacete;

Ochoa Parra N, Hospital Universitario 12 de Octubre, Madrid;

Otero Candelera R, Hospital Universitario Virgen del Rocío, Sevilla;

Otero González I, Hospital Universitario A Coruña, A Coruña;

Pastor Pérez F, Hospital Clínico Universitario Virgen de la Arrixaca, Murcia;

Pérez Peñate GM, Hospital Universitario de Gran Canaria Dr. Negrín, Las Palmas;

Pérez Sagredo J, Hospital El Bierzo, León;

Pérez L, Hospital Universitario Central de Asturias, Asturias;

Ramírez Martín P, Hospital Universitario Nuestra Señora de Candelaria, Santa Cruz de Tenerife;

Ramón Capilla M, Hospital General Universitario de Valencia, Valencia;

Recio Mayoral A, Hospital Universitario Virgen Macarena, Sevilla;

Rey Chacón I , Hospital Universitario Virgen del Rocío, Sevilla;

Ribas Sola J, Hospital Universitari de Bellvitge, Barcelona;

Rodríguez Chiaradía DA, Hospital del Mar, Barcelona;

Rodríguez Penas D, Complejo Hospitalario Universitario Santiago de Compostela, A Coruña;

Rueda Soriano J, Hospital Universitario y Politécnico La Fe, Valencia;

Sáez Giménez B, Hospital Universitario Vall d´Hebrón, Barcelona;

Safont B , Hospital Clínico Universitario de Valencia, Valencia;

Sala Llinas E, Hospital Universitario Son Espases, Islas Baleares;

Segovia Cubero J, Hospital Universitario Puerta de Hierro - Majadahonda, Madrid;

Sintes Permanyer H, Hospital Universitari Germans Trias i Pujol, Barcelona;

Soto Abánades C, Hospital Universitario La Paz, Madrid;

Suberviola V, Hospital Universitario Infanta Leonor, Madrid;

Tenes JA, Hospital Universitario Ramón y Cajal, Madrid;

Torrents Vilar A, Hospital General Universitario de Castellón, Castellon;
